# Stapled versus hand-sewn anastomoses after bowel resection in patients with crohn disease

**DOI:** 10.1186/s12893-024-02410-6

**Published:** 2024-05-03

**Authors:** Saleh Lahes, Celine Fischer, Dorian Igna, Peter Jacob, Matthias Glanemann

**Affiliations:** 1 Department of Surgery, University Hospital Knappschaftskrankenhaus Bochum, Ruhr- University Bochum, In der Schornau 23-25, Bochum, Germany; 2 Department of General, Visceral, Vascular and Pediatric Surgery, Saarland University, Homburg/Saar, Germany

**Keywords:** Crohn Disease, Stapler Versus Hand-Sewn Anastomosis, Colorectal surgery, Postoperative complications

## Abstract

**Background:**

Anastomosis configuration is an essential step in treatment to restore continuity of the gastrointestinal tract following bowel resection in patients with Crohn’s disease (CD). However, the association between anastomotic type and surgical outcome remains controversial. This retrospective study aimed to compare early postoperative complications and surgical outcome between stapler and handsewn anastomosis after bowel resection in Crohn’s disease.

**Methods:**

Between 2001 and 2018, a total of 339 CD patients underwent bowel resection with anastomosis. Patient characteristics, intraoperative data, early postoperative complications, and outcomes were analyzed and compared between two groups of patients. Group 1 consisted of patients with stapler anastomosis and group 2 with handsewn anastomosis.

**Results:**

No significant difference was found in the incidence of postoperative surgical complications between the stapler and handsewn anastomosis groups (25% versus 24.4%, *p* = 1.000). Reoperation for complications and postoperative hospital stay were similar between the two groups.

**Conclusion:**

Our analysis showed that there were no differences in anastomotic leak, nor postoperative complications, mortality, reoperation for operative complications, or postoperative hospital stay between the stapler anastomosis and handsewn anastomosis groups.

## Introduction

Crohn’s disease (CD) is a chronic inflammatory bowel disease which can affect any segment of the gastrointestinal tract from the mouth to the anus, with most cases involving the terminal ileum and the coecum [1, 2].

Medical therapy for CD has progressed considerably; however, surgery plays an important role in the treatment of CD, especially to case of failure of medication or of CD-related complications, such as stricture, abscess, fistula, perforation, or malignant transformation [3].

The presence of fistulas or perforations was shown to be associated with a higher rate of complications than other indications [4, 5].

Stapled and handsewn anastomoses are universally used to restore continuity of the intestinal tract after intestinal resection.

Stapling devices have been used since the late 1970s. Few studies have reported the anastomotic technique in Crohn’s disease [6–12]. The role of the surgical technique in performing anastomosis in the postoperative results has been an issue of constant debate. Several studies have examined different surgical techniques to perform anastomosis in CD patients to find a method that minimizes perioperative morbidity, including bowel obstruction, wound infection, anastomotic leak, and operative time, as well as recurrent Crohn’s disease activity at the site of anastomosis. Unfortunately, most of these studies are controversial and either do not demonstrate a significant difference or report conflicting results.

A meta-analysis in 2007, which compared handsewn end-to-end and stapled side-to-side anastomoses with regard to early postoperative complications in Crohn’s disease patients, did not show a specific benefit for either technique [10].

McLeod et al. published results from a prospective randomized trial of 170 patients. They did not find any difference in early postoperative complication rates, such as anastomotic leak and wound infection rates, in either anastomotic technique [11].

A randomized controlled multicenter trial in 2012 observed no difference between the two types of anastomoses in terms of postoperative complications [12]. In a meta-analysis conducted in 2018, Feng et al. showed no significant difference in complications such as anastomotic leak, wound infection, postoperative hospital stays, and mortality, but they observed that overall postoperative complications, clinical recurrence, and reoperation a more likely to be superior for stapled anastomosis than for handsewn anastomosis [13].

The aim of this retrospective study was to compare the incidence of postoperative complications in CD patients undergoing stapler anastomosis with those undergoing handsewn anastomosis.

## Patients and methods

We analyzed patients who underwent abdominal surgery for CD between 2001 and 2018 in the Department of General, Visceral, Vascular, and Pediatric Surgery at the University Hospital of Saarland. Patients who underwent anastomosis were included in this study, whereas all patients without any intestinal, colon, or rectal anastomosis were excluded. Only histologically confirmed CD patients were included in the analysis. The patients required small bowel or colorectal resection, ileostomy closure, or colostomy closure.

Anastomoses were performed using the stapler (stapled group) or handsewn anastomoses (handsewn group). Thirty-six patients underwent stapled functional end-to-end, end-to-side, side-to-end, or side-to-side anastomoses.

Handsewn anastomoses were performed in 303 patients. A single-row or double-row anastomosis was constructed in this group. The handsewn anastomoses were sutured end-to-end, end-to-side, side-to-end, or side-to-side.

Demographics and relevant preoperative data were extracted from patient charts, including sex, age, body mass index (BMI), American Society of Anesthesiologists score (ASA), immunosuppressive medication for CD, history of previous surgical procedures, disease duration, Charlson Comorbidity Index (CCI), indication for surgery, and preoperative complications.

Preoperative complications of CD were defined by the presence of an intraabdominal abscess, fistula (enterovesical, enterovaginal, enterocutaneous, or enteroenteric), or bowel perforation as an indication for abdominal surgery. Other indications for abdominal surgery, such as bowel obstruction, intestinal stricture, medication-refractory disease, and elective closure of a stoma, were also included in the study.

Preoperative medical treatment protocols with mesalazine, corticosteroids, immunomodulators (azathioprine and 6-mercaptopurine), or anti-TNF-α antibodies have been documented.

Intraoperative characteristics were recorded as elective or emergency surgery, median duration of operation, median blood loss, and anastomosis location.

Postoperative surgical complications, non-surgical complications, mortality, duration of hospital stay, and reoperations for complications were recorded for the groups during the postoperative 30 days.

The following outcome used to compare the stapled group with the handsewn group:

The incidence of postoperative surgical complications, overall complications, thirty-day morbidity and mortality, postoperative hospital stay, and reoperation for complications.

Surgical complications included wound infection, anastomotic leakage, intraabdominal abscess, enterocutaneous fistula, bleeding, peritonitis, and sepsis. Overall complications included surgical complications along with other complications such as cardiac (cardiac arrest, myocardial infarction, atrial or ventricular dysrhythmia requiring treatment), pulmonary (pneumonia, pleural effusion, pleural empyema, pneumothorax, pulmonary edema, atelectasis, acute respiratory distress syndrome, and respiratory insufficiency), gastrointestinal (paralysis, GI bleeding), neurological (apoplex, postoperative delirium), nephrological (acute renal insufficiency), and urological (urinary tract infection) complications.

### Statistics

Statistical analysis was performed using SPSS® version 20.0 software (IBM, Armonk, New York, USA). Categorical data are presented as absolute numbers and percentages.

Fisher’s exact test was used to compare the groups of categorical variables. The Mann-Whitney U test was used to compare groups of continuous variables. Statistical significance was set at *p* < 0.05.

## Results

A total of 339 patients (161 men and 188 women) were included in this study. Indications for surgery were medication-refractory disease, CD-related complications such as intestinal stricture, intestinal perforation, intraabdominal abscess, or fistula and elective closure of an ileostomy or closure of a colostomy. The patients were divided into two groups: stapler anastomosis (*n* = 36) and handsewn anastomosis (*n* = 303).

The patient characteristics are listed in Table 1. Sex, age, body mass index (BMI), American Society of Anesthesiologists score (ASA), and Charlson Comorbidity Index (CCI) were similar in both groups, with no significant differences. There was no difference in the number of patients receiving immunosuppressive therapy in the stapled group compared with the sutured group (75% vs. 79.5%, *P* = 0.519). The number of patients who had previously undergone abdominal surgery was also similar in both the groups. The median duration of symptoms before surgery was 8.5 years in the stapled group, compare with 10 years of the patients in the sutured group.

**Table 1:** Demographic and preoperative data

	Stapler anastomosis *n* = 36	Handsewn anastomosis *n* = 303	*p*-value
Male/female ratio	20/16	141/162	0.378
Median Age (years) range	35.5 (15–73)	40 (16–76)	0.138
BMI, median, range	22.9 (14.2–34)	23.4 (14.2–43.1)	0.639
ASA			0.114
I II III IV	3 30 3 0	13 242 45 3	
Charlson Comorbidity Index (CCI)			0.465
0 1 2 3 4 5 6 7 8 12	25 (69.4%) 5 (11.1%) 0 2 (5.6%) 1 (2.8%) 1 (2.8%) 1 (2.8%) 0 (0%) 1 (2.8%) 0 (0%)	189 (62.4%) 40 (13.2%) 31 (10.2%) 13 (4.3%) 10 (3.3%) 10 (3.3%) 5 (1.7%) 3 (1%) 1 (0.3%) 1 (0.3%)	
Immunosuppressive medication Yes No	27 (75.0%) 9 (25.0%)	241 (79.5%) 62 (20.5%)	0.519
Previous abdominal surgery			1.000
Yes No	27 (75%) 9 (25%)	222(73.3%) 81 (26.7%)	
Median duration of symptoms before operation (range), years	8.5 (0–22)	10 (0–41)	0.158
Preoperative complication			0.41
Yes No	10 (27.8%) 26 (72.2%)	123 (40.6%) 180 (59.4%)	
Abscess Fistula Perforation	3 (8.3%) 8 (22.2%) 0 (0%)	29 (9.6%) 91 (30%) 24 (7.9%)	1.000 0.438 0.091

The Fisher’s exact test and Mann–Whitney U test were used. Categorical data were presented as absolute numbers and percentages

The indications for surgery were similar between the groups. Preoperative complications such as abscess, fistula, or perforation were found in 27.8% of the patients in the stapled group compared to 40.6% of the patients in the sutured group.

Table 2 provides an overview of the operative details of the two groups. There was no significant difference between the two groups in terms of elective surgery, emergency surgery, duration of operation time, or blood loss. In terms of location, we found a significant difference between the two groups in ileocolic and colorectal anastomoses.

**Table 2:** Intraoperative Date

	Stapler anastomosis n:36	Handsewn anastomosis n:303	*p*-value
Electiv OP Emergency OP	35 (97.2%) 1 (2.8%)	262 (86.5%) 41 (13.5%)	0.065
Median Duration of operation (min), range	129 (55–298)	125 (35–380)	0.404
Median blood lost in ml (range)	100 (0-2000)	50 (0-2000)	0.087
Location of Anastomosis			
Small bowel Ileocolic Ileorectal Colocolic Colorectal	4 (11.4%) 16 (47.5%) 2 (5.6%) 2 (5.6%) 12 (33.3%)	38 (12.5%) 243 (80.2%) 17 (5.6%) 11 (3.6%) 13 (4.3%)	1.000 < 0.001 1.000 0.637 < 0.001

Fisher’s exact test and Mann–Whitney U test were used

The most performed anastomosis in the stapled group was the ileocolic anastomosis in 16 patients (47.5%), followed by colorectal in 12 patients (33.3%). In the sutured group the ileocolic anastomosis was performed in 243 patients (80.2%), followed by small bowel anastomosis in 38 patients (12.5%).

Table 3 illustrates the postoperative complications, thirty-day morbidity and mortality, postoperative hospital stay, and reoperation for complications in both the groups.

**Table 3:** Postoperative data and complications

	Stapler anastomosis, n:36	Hand sewn anastomosis, n:303	*p*-value
Surgical complications, n (%)	9 (25%)	74 (24.4%)	1.000
Wound infection	5 (13.9%)	55 (18.2%)	0.648
Anastomotic leak	3 (8.3%)	24 (7.9%)	1.000
Intra-abdominal abscess	2 (5.6%)	12 (4%)	0.651
Enterocutaneous fistula	2 (5.6%)	8 (2.6%)	0.288
Bleeding	2 (5.6%)	7 (2.3%)	0.246
Peritonitis	1 (2.8%)	7 (2.3%)	0.597
Sepsis	0 (0%)	2 (0.7%)	1.000
Overall complication	10(27.8%)	114 (37.6%)	0.277
Cardiac	0 (0%)	2 (0.6%)	1.000
Pulmonary	0 (0%)	13 (3.8%)	0.375
Gastrointestinal	0 (0%)	23 (7.6%)	0.151
Neurological	2 (5.6%)	3 (1%)	0.089
Nephrolgical	0 (0%)	4 (1.3%)	1.000
Urological	0 (0%)	8 (2.6%)	1.000
Mortality, n	0 (0%)	2 (0.7)	1.000
Median duration of postoperative hospital stay (range), days	11 (3–60)	12 (4-164)	0.781
Reoperation for complication	9 (25%)	58 (19.1%)	0.383

Fisher’s exact test and Mann–Whitney U test were used. Categorical data are presented as absolute numbers and percentages

There was no significant difference in postoperative surgical complications between the two groups (25% vs. 24.4%). The most common surgical complication was wound infection (13.9% vs. 18.2%), followed by anastomotic leak (8.3% vs. 7.9%).

The incidence of other surgical complications, including abscess, fistula, bleeding, peritonitis, and sepsis, was comparable between the both groups.

The incidence of overall complications in the stapled and sutured groups was 27.8% and 37.6%, respectively. No significant differences were observed between the groups.

The incidence of non-surgical complications such as cardiac, pulmonary, gastrointestinal, neurological, nephrological, and urological complications was comparable between the two groups. There were no significant differences between the groups.

Although mortality was reported in 2 patients in the sutured group, a significant difference between the groups was not detected. Mortality is attributed to pneumonia, sepsis, and multiple-organ failure.

The median duration of postoperative hospital stay in the stapled group was 11 days, whereas that in the sutured group was 12 days. No significant differences were observed (*P* = 0.781).

In the stapled group, all patients with surgical complications underwent reoperation (25%). 19.1% of the patients in the sutured group required reoperation. In both groups, no significant differences were observed (*p* = 0.383).

Table 4 illustrates the postoperative surgical complications and the location of anastomosis between the both groups. Although there were significant differences between the anastomotic localization in the ileocolic and colorectal anastomosis (*P* < 0.001) between both groups, it was no significant difference in the postoperative complications ( *P* = 0,542, *P* = 0.378).

**Table 4:** Postoperative surgical complication and location of anastomosis

Location of Anastomosis	Stapler anastomosis		Handsewn anastomosis		*p*-value n	*p*-value complication
	n:36	Postoperative surgical complication	n:303	Postoperative surgical complication		
Small bowel	4 (11.4%)	0 (0%)	38 (12.5%)	13(34.2%)	1.000	0.293
Ileocolic	16 (47.5%)	5 (31,3%)	243 (80.2%)	56 (23%)	< 0.001	0.542
Ileorectal	2 (5.6%)	0 (0%)	17 (5.6%)	4 (23.5%)	1.000	1.000
Colocolic	2 (5.6%)	1 (50%)	11 (3.6%)	3 (27.3%)	0.637	1.000
Colorectal	12 (33.3%)	2 (16.7%)	13 (4.3%)	5 (38.5%)	< 0.001	0.378

Fisher’s exact test was used. Categorical data are presented as absolute numbers and percentages

## Discussion

The incidence and prevalence of CD are rapidly increasing worldwide, and many patients with CD eventually require surgical intervention. Approximately 70–90% of patients with CD undergo surgical treatment during their lifetime, namely ileocolectomy in the majority of cases [14–17]. Postoperative complications, recurrence, and even reoperation can occur because of the recurrent pathophysiology of the disease. Surgery plays an important role in CD management. Many observations support the hypothesis that anastomosis plays an important role in the outcome after resection. The technique of anastomosis is controversial in the literature. We conducted a retrospective study to compare the different anastomotic configurations after intestinal resection in patients with CD. A total of 339 patients were included in this retrospective analysis. Handsewn anastomosis was performed in the majority of patients (303) and stapler anastomosis was performed in 36 patients.

In this study, we primarily compared all anastomotic configurations after bowel resection for patients with CD, end-to-end, end-to-side, side-to-end or side-to-side anastomosis, including one single row anastomosis or double row in the hand sewn anastomosis.

The preoperative characteristics and intraoperative factors were similar between both groups. There was a significant difference in the anastomosis location between the two groups; ileocolic anastomosis was performed in 80.2% of the handsewn group versus 47.5% of the stapler group (*p* < 0.001). The colorectal anastomosis was performed more in stapler group (33.3% versus 4.3%, *p* < 0.001).

The results of this study revealed that both techniques offered similar results in terms of postoperative complication, thirty-day morbidity and mortality, postoperative hospital stay, and reoperation for complications.

In our analysis, there was no significant difference in the rate of postoperative complications between stapler and handsewn anastomoses. In accordance with our results, a meta-analysis from 2007 including 661 patients, which compared handsewn end-to-end and stapled side-to-side anastomoses with regard to early postoperative complications in Crohn’s disease patients, reported no specific benefit for either technique [10]. In the same line of evidence, prospective randomized trial with 170 patients conducted by McLeod et al. found no significant difference in postoperative complication rates, such as anastomotic leak and wound infection rates, between both anastomotic techniques [11]. They reported an anastomotic leak rate of 7% in both groups and a postoperative wound infection rate of 9% vs. 11% (side-to-side vs. end-to-end anastomosis). A randomized controlled trial induced by Zurbuchen et al. observed no difference between handsewn end-to-end and side-to-side stapler anastomosis [12], comparable to our results, too.

A meta-analysis from 2018 compared anastomotic techniques. There was no significant difference in complications such as anastomotic leak, wound infection, postoperative hospital stay and mortality between the groups. They found a higher superiority for stapled anastomosis than for handsewn anastomosis due to clinical recurrence [13].

In contrast, a meta-analysis conducted in 2014 by He et al. concluded that stapled side-to-side anastomosis would appear to be the preferred procedure after ileocolic resection for CD, with reduced overall postoperative complications, especially anastomotic leak, and decreased recurrence and reoperation rates [18]. The authors saw limitations to this meta-analysis: the number of eligible randomized controlled trials was small (3 randomized control trials, 1 prospective non-randomized study, and 4 retrospective studies). Two randomized controlled trials of this meta-analysis observed no significant differences in early postoperative complications. A retrospective study of 122 patients induced by Resegotti et al. found that side-to-side stapled anastomosis after ileocolic resection reduced anastomotic leak rates and postoperative hospital stay in Crohn’s disease surgery compared to handsewn anastomosis [19].

A retrospective study conducted in 1999 with 123 patients suggested that stapled functional end-to-end ileocolonic anastomosis is associated with a lower incidence of complications and that early anastomotic recurrence is less common than that after sutured end-to-end anastomosis [20].

In 2011, a functional end-to-end hand-sewn antimesenteric Kono-S anastomosis was described for the first time in Crohn’s disease patients. It demonstrated a lower surgical recurrence rate than the standard technique, but no difference in postoperative complications [21]. In our study, Kono-S anastomosis was not performed.

Although there is no difference in terms of postoperative morbidity regardless of type of anastomosis, high-volume centers have lower postoperative mortality and 30-day readmission rates for patients undergoing abdominal surgery for CD. CD patients especially with complex procedures may benefit from specialized centers [22, 23].

The data from our study can be criticized because they were retrospective, non-randomized groups. There may have been a selection bias, the patients were not randomized into the two treatment groups. Another limitation of our study is the lack of ability to record Crohn’s recurrences with a longer follow-up interval.

Despite these limitations, the strength of this study is that the anastomosis technique was performed in different bowel locations. Most studies have compared patients with ileocolic anastomosis only. Our study included all Crohn disease patients that underwent anastomosis, including those with preoperative complications like abscesses, fistulas, and conglomerate tumors. There was no selection of patients, so our results are representative.

## Conclusion

Our study showed that there were no differences in anastomotic leak, other postoperative complications, mortality, reoperation for operative complications, and postoperative hospital stay between the stapler anastomosis and handsewn anastomosis groups. Further randomized controlled trials with extended follow-up are needed to compare postoperative results, recurrence, and reoperation rates between stapler and handsewn anastomosis.

## Data Availability

The datasets generated during and/or analyzed during the current study are not publicly available, but are available from the corresponding author on reasonable request.The need for informed consent was waived by the ethics committee/Institutional Review Board of The Medical Ethics Committee of the Medical Association of Saarland, Germany(file number: 231/20) , because of the retrospective nature of the study.
